# Characteristic Evaluation of Graphene Oxide for Bisphenol A Adsorption in Aqueous Solution

**DOI:** 10.3390/nano6070128

**Published:** 2016-07-02

**Authors:** Thatchaphong Phatthanakittiphong, Gyu Tae Seo

**Affiliations:** 1 Department of Eco-friendly Offshore Plant FEED Engineering, Changwon National University, 20 Changwondaehak-ro, Uichan-gu, Changwon 51140, Korea; thatchaphong@changwon.ac.kr; 2 Department of Environmental Engineering, Changwon National University, 20 Changwondaehak-ro, Uichan-gu, Changwon 51140, Korea

**Keywords:** adsorption characteristics, bisphenol A, graphene oxide, hydrogen bonding, oxygen-containing functional groups, π–π interactions

## Abstract

This paper investigates the characteristics of graphene oxide (GO) for Bisphenol A (BPA) adsorption in water. Batch experiments on the influence of significant parameters were performed. While an improvement of the adsorption capacity of BPA was obtained by the increment of contact time and the initial BPA concentration, the increment of pH above 8, GO dosage, and temperature showed the reverse results. The thermodynamic study suggested that BPA adsorption on GO was an exothermic and spontaneous process. The kinetics was explained by the pseudo-second-order model which covers all steps of adsorption. The fit of the results with the Langmuir isotherm indicated the monolayer adsorption. At 298 K, the adsorption reached equilibrium within 30 min with the maximum adsorption capacity of 49.26 mg/g. The low BPA adsorption capacity of GO can be interpreted by the occurrence of oxygen-containing functional groups (OCFGs) that are able to form hydrogen bonds with the surrounding OCFGs and water molecules. This effect inhibited the role of π–π interactions that are mainly responsible for the adsorption of BPA.

## 1. Introduction

Nanomaterials have been implemented in a variety of applications for water treatment. Their attractiveness can be attributed to their extraordinary properties, such as high surface area for adsorption, high activity for (photo) catalysis, antimicrobial properties for disinfection and biofouling control, superparamagnetism for particle separation, and other unique optical and electronic properties that find use in novel treatment processes and sensors for water quality monitoring. Additionally, several studies on water treatment technology showed promising benefits of applying nanomaterials in terms of their performance, cost effectiveness, and environmental acceptability [1,2,3]. In the adsorption process, carbon nanomaterials (CNMs), well-known adsorbents for water treatment with high specific surface area, have recently received close attention from many researchers for the adsorption of various kinds of aquatic pollutants, including endocrine-disrupting compounds (EDCs). Graphene oxide (GO), a carbon-based hexagonal structure decorated with the largest, as compared to other CNMs, proportion of oxygen-containing functional groups (OCFGs), has proved to be a remarkable candidate for the adsorption of various contaminants in water, including heavy metals (Cd^2+^, Zn^2+^, Pb^2+^, Cu^2+^, Cr^6+^, etc.), dyes (methyl blue, methyl violet, rhodamine B, orange G, etc.), and pharmaceutical antibiotics (tetracycline, oxytetracycline, doxycycline, etc.). The large constituent of OCFGs (hydroxyl, carbonyl, carboxyl, epoxy, and alkoxy) provides a strong negative charge for GO, activating the electrostatic interactions with the positively-charged adsorbates. These interactions also comprise the hydrogen bonding between the hydrogen atom and the highly electronegative atoms (oxygen, nitrogen, and fluorine). Furthermore, similarly to other CNMs, GO is also composed of sp^2^ hybridized benzene rings that are able to build π–π interactions with the benzene rings of adsorbate, and even to create the cation–π bonding with metal ions/protonated amino groups. This unique capacity promotes the excellence of GO as a novel adsorbent for the removal of aquatic contaminants. Another advantage of this material is that it can be synthesized from low-cost graphite [4].

Bisphenol A (BPA, C_15_H_16_O_2_), a phenolic compound, is considered to be one of EDCs that can cause harmful effects on human and wildlife endocrine systems, even at extremely low concentrations. This compound is widely used as a component in industrial products, such as plastic, food cans, epoxy resin, and flame retardants. It is a major contaminant in the municipal wastewater and can also be extensively found in industrial wastewater, sewage treatment effluent, ground water, surface water, landfill leachate, blackish water, and seawater [5,6,7]. The pathways through which BPA gets from these water sources to the human body are possibly from drinking water and reuse water for agricultural purposes [8]. In order to cut off these pathways, a proper, economically-viable treatment process that can ensure a highly-efficient removal of BPA is urgently required.

Cortés-Arriagada et al. [9] investigated the adsorption of BPA on graphene and GO using the density functional theory (DFT) method. At the basal plane of GO, the highest adsorption energy was obtained from the binding between BPA and hydroxyl groups. At the edges of GO, valuable energies were obtained from two cases (BPA–carbonyl groups and BPA–carboxyl groups), while the outcome of the binding with hydroxyl groups was insignificant. The adsorption energies were found to be higher at the basal plane as compared to the edge, which may be explained by the support of π–π interactions. However, from the comparison of the adsorption energies in both adsorbents, it can be inferred that the adsorption energies of BPA onto GO are either higher than, or comparable to, that of BPA onto graphene. The resembling consequence was also reported by Jin et al. [10]. These computational studies provide valuable evidence in support of the use of GO as an effective adsorbent for the adsorption of BPA. By contrast, the results gathered from experimental studies remain inconclusive. The studies on the adsorption of some aromatic compounds (including BPA) that definitely have no positive charge in the molecules showed that the use of GO is ineffective. Specifically, it was demonstrated that the adsorption of these compounds is strongly induced by π–π interactions, rather than by the electrostatic interactions, including hydrogen bonding. Thus, the functional groups on GO seem to hinder π–π interactions. This conclusion is consistent with several previous studies that observed the enhancement of adsorption efficiency after the reduction of GO. The reduction process will eliminate OCFGs and restore the sp^2^-hybridized structure that improves the potential of π–π interactions [11].

Since, considering the discrepancy between the results of computational and experimental studies, the adsorption behavior of BPA onto GO remains questionable, further research is needed to resolve this confusion.

In the present study, we experimentally investigated the GO adsorption of BPA. To this end, the fundamental examinations including kinetic and isotherm models were examined. The effects of various factors (such as contact time, initial BPA concentration, GO dosage, pH, and temperature) on the removal efficiency were systematically explored. Finally, the evaluation of the experimental results was performed to get a clear understanding of the adsorption characteristics.

## 2. Results

### 2.1. Characterization of Synthesized Graphene Oxide

The results of the elemental analysis of the adsorbents by SEM/EDS are presented in Table 1. The ratio between the percentages of carbon and oxygen (C/O ratio) represents the proportion of OCFGs on the materials. The lowest value of this ratio in GO, as compared to other CNMs, implies the richest content of OCFGs. The C/O ratio of our synthesized GO excellently coheres with the results reported in previous studies.

**Figure 1:**
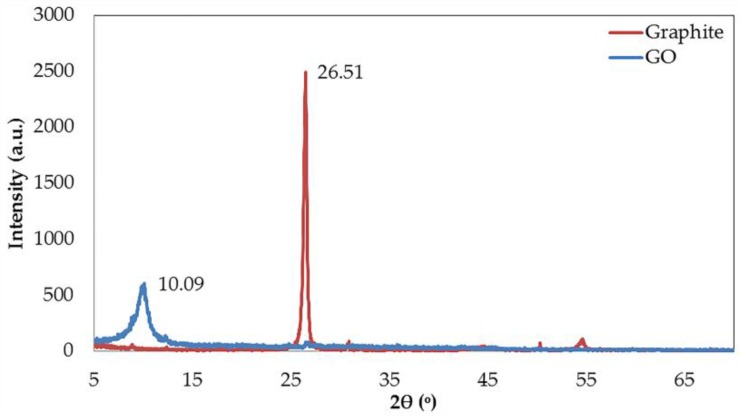
The powder X-ray diffraction (XRD) patterns of graphite and GO.

An important evidence needed to assure the completion of synthesized GO is the XRD analysis. The XRD patterns of graphite and synthesized GO are shown in Figure 1. The diffraction peaks of graphite and GO appeared at 26.51° and 10.09°, revealing the interlayer spacing of 0.34 nm and 0.88 nm, consecutively. The increment of spacing after the oxidation of graphite demonstrates the intercalation of OCFGs between the layers of graphite. This result is also congruent with the XRD data of GO reported elsewhere [16].

### 2.2. Parameters for BPA Adsorption on Graphene Oxide

#### 2.2.1. Effect of Contact Time and Initial BPA Concentration

**Figure 2:**
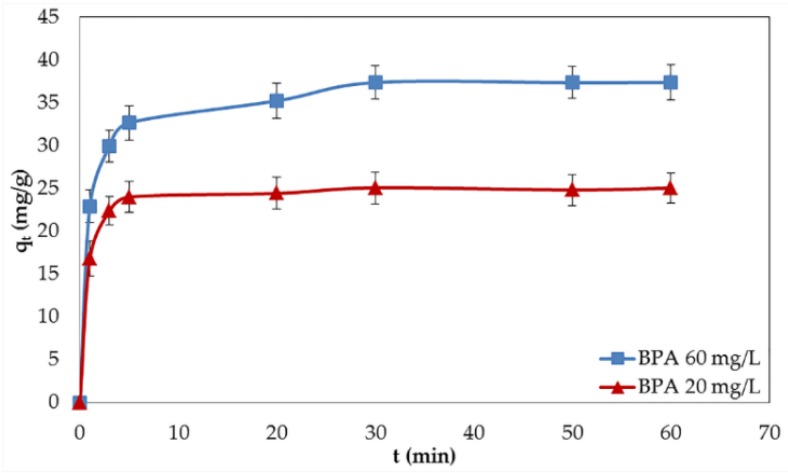
Effect of contact time and initial concentration on the adsorption of bisphenol A (BPA) by GO. Error bars represent the standard deviations of three replicates. q_t_: The amounts of BPA adsorbed on GO at time interval t (mg/g).

The study of the effect of contact time is compulsory for minimizing the operation time of the treatment process which may promote the economic benefits to the system. Figure 2 shows the effect of contact time on the adsorption of BPA by GO. The rapid adsorption occurred significantly during the first 5 min and gradually diminished until reaching equilibrium within 30 min, after which the adsorption capacity did not increase anymore. The rapid adsorption at the initial stage could be interpreted by the large number of available active sites and high driving force, both of which make BPA swiftly transfer to GO. Shortly afterwards, the subsidence in the adsorption rate was observed, which could be ascribed to the fewer available adsorption sites and the decrease in the driving force [17]. As compared to CNTs and other porous materials, the contact time required for GO to reach the adsorption equilibrium is very short [18]. This characteristic may relate to the unique morphology of GO (flat and single sheet) that guarantees the adequate number of active sites on the surfaces. According to the rapid kinetics, the use of GO is beneficial for water treatment systems in terms of efficiency and economy by reducing the required reactor volumes [19]. Although the contact time of 30 min is sufficient to reach the adsorption equilibrium, further research was performed using the contact time of 2 h to ensure that the reaction equilibrium would be achieved.

An important factor that affects the adsorption kinetics is the initial concentration of the adsorbate. Figure 2 presents the relationship between adsorption capacity and contact time at different BPA concentrations. As can be seen in Figure 2, the adsorption capacity of BPA increased with the increase of the initial BPA concentration. This tendency could be explained by a larger driving force provided by the higher BPA concentration, which could defeat the mass transfer resistance between the aqueous and solid phases [20].

#### 2.2.2. Effect of GO Dosage

**Figure 3:**
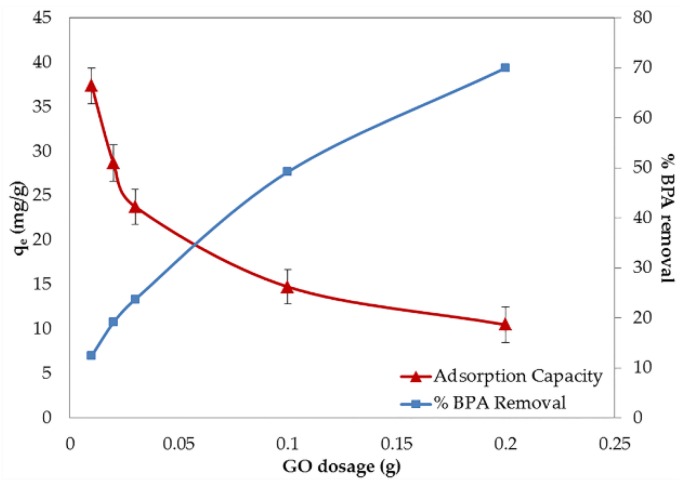
Effect of the GO dosage on the adsorption of BPA. q_e_: the amounts of BPA adsorbed on GO at equilibrium (mg/g).

Recently, the issue of cost-effectiveness has started to be seriously considered for the suitable design of water treatment systems. The optimization of the adsorbent dosage used in the system is a crucial aspect for saving material costs. The effect of adsorbent dosage on the removal of BPA and the adsorption capacity are shown in Figure 3. After increasing the GO dosage from 10 mg to 200 mg, a distinct augmentation of the BPA removal efficiency up to 57% was observed. This result may be explained by the enhancement of the surface area and the number of available active sites in the adsorbent after the supplement of the GO dosage [21]. On the other hand, a 72% decrement in the adsorption capacity was obtained after increasing the GO dosage. This observation may be attributed to the excess of the active sites of the adsorbent, as compared to the saturated threshold adsorption points, which lead to a partial occupation of the active sites by the BPA molecules. Furthermore, in the circumstances of high solid content, the overlapping and aggregation (particle–particle interactions) of the solid that can block the adsorption sites are possibly built and the electrical surface charges of the adjacent particles cause a decline in the adsorption efficiency. From these results, 10 mg of GO showed the highest adsorption capacity. Thus, this dosage was applied in further experiments in the present study. A similar decision was also reported in an adsorption study of other adsorbates [20].

#### 2.2.3. Effect of pH

**Figure 4:**
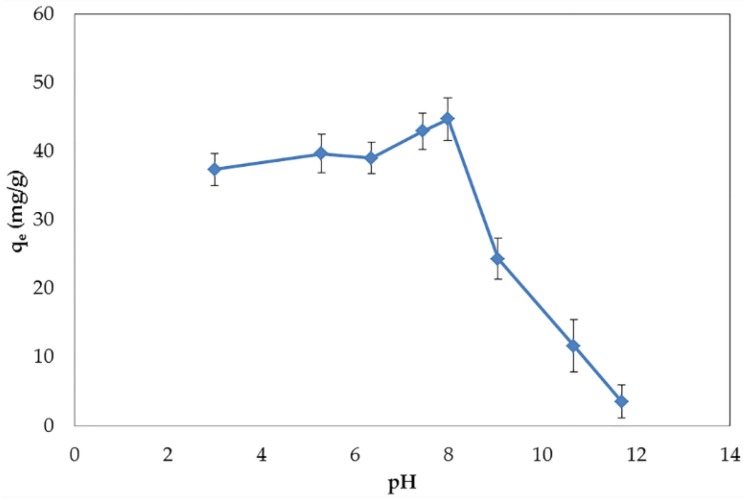
Effect of pH on the adsorption capacity of BPA.

The pH of solution is an important parameter to consider. This parameter can affect both the electrical surface charges of the adsorbent and the dissociation of the adsorbate. The effect of pH differs depending on the type of adsorbents and the dissociation constants (P_Ka_) of the adsorbate. In water treatment systems, the pH of influent water varies according to the sources of water and amounts to 4.8–9.5 in surface water, 6.7–9.5 in ground water, 7.9–8.3 in seawater, 7.2–7.4 in domestic wastewater, and 5.0–8.3 in leachate water [6,22,23,24,25,26,27]. According to this wide range of pH, the understanding of the effect of pH on adsorption characteristics is required. The influence of pH on the adsorption of BPA by GO is shown in Figure 4. In the acidic pH range, the adsorption capacity was stable from pH 3 to 6 and gradually increased at neutral pH. It may be explained by the abatement of hydrogen ions after the increase of pH, which reduces the chance of competition between hydrogen ions and the BPA molecules. Due to the natural negative charge of GO over the whole pH range, it is possible for hydrogen ions to be adsorbed by GO through electrostatic interactions [4]. By contrast to the basic pH range, the adsorption capacity decreased dramatically at pH above 8. This severe reduction could be described by the P_Ka_ of BPA (9.6–10.2). In aqueous solutions, the adsorbates will stay in their molecular form at pH lower than P_Ka_ and will lose their protons at pH above P_Ka_. Thus, at pH around or higher than P_Ka_, the BPA molecules are deprotonated to bisphenolate anions (HBPA^−^ and BPA^2−^) and cause the electrostatic repulsion with negatively-charged GO [28]. Our results cohere with the adsorption of BPA onto Fe_3_O_4_/GO and other adsorbates reported in previous studies [28,29,30].

#### 2.2.4. Effect of Temperature and Thermodynamic Study

**Figure 5:**
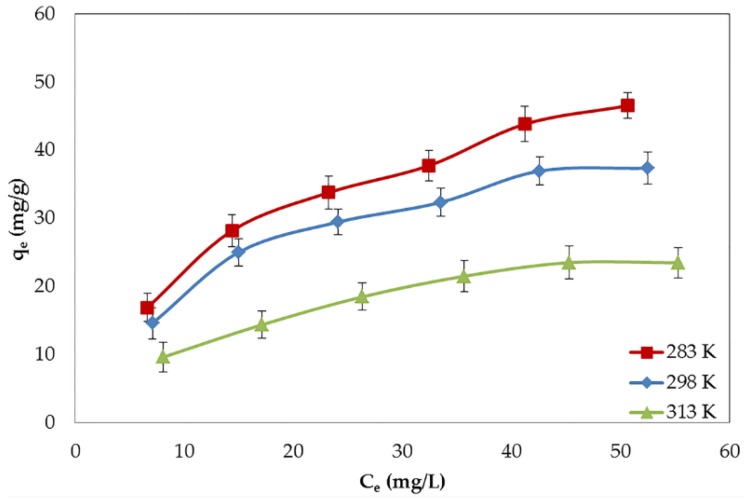
Effect of temperature on the adsorption of BPA by GO.

Temperature is a crucial parameter for studying adsorption characteristics. The adsorption process can be categorized into physical adsorption (physisorption) and chemical adsorption (chemisorption). For chemisorption, the contaminants are adsorbed onto the surface of the adsorbent through chemical bonding. To accomplish this type of adsorption, the heat of adsorption should be sufficiently high to reach the energy of chemical bonds. Thus, the chemisorption is effective at high temperatures [31]. On the other hand, the physisorption is held by the weak van der Waals force where a low temperature is preferable [32]. To classify the type of the adsorption process, the study of the effect of temperature and thermodynamics is essential. The effect of temperature on the adsorption of BPA by GO was evaluated (see Figure 5). The maximum uptake of BPA was found to decrease from 62.89 mg/g to 32.90 mg/g with the increase of the temperature from 283 K to 313 K. This finding suggests that the BPA adsorption on GO is an exothermic process which is activated more at low temperatures.

Even though the BPA adsorption on GO can be concluded to be an exothermic process, a thermodynamic study is needed to assure the type of the adsorption process. The analysis can illustrate the adsorption process from the aspect of energy change. The thermodynamic parameters, such as standard enthalpy change (ΔH^0^, kJ/mol), standard entropy change (ΔS^0^, J/mol/K), and standard Gibbs free energy change (ΔG^0^, kJ/mol), could be calculated using Equations (1) and (2):

(1) $${\text{lnK}}_{d}=\frac{\Delta S^{0}}{R}+\frac{\Delta H^{0}}{\text{RT}}$$

(2) $$\Delta G^{0}=\Delta H^{0}-T\Delta S^{0}$$

where K_d_ (L/g) is the distribution coefficient; R (8.314 J/mol/K) is universal gas constant.

To calculate the thermodynamic parameters, first, the K_d_ value is derived from the value of *y*-axis intercept of the linear plot between (q_e_/C_e_) versus q_e_ at different temperature. In the next step, a linear graph is plotted between lnK_d_ versus 1/T × 10^3^ where T is temperature (K). Then, the values of ΔH^0^ and ΔS^0^ can be consecutively calculated from the slope and the value of *y*-axis intercept. Table 2 presents the thermodynamic parameters calculated using the above-mentioned method. The negative values of ΔG^0^ at all temperatures indicate that the GO sorption of BPA was a thermodynamically feasible, spontaneous process. At the same time, the increase of ΔG^0^ values after the temperature decrease indicates a more efficient adsorption at a lower temperature. Moreover, for the physisorption process, ΔG^0^ values are generally within the range between 0 and −20 kJ/mol, while the variation between −80 and −400 kJ/mol is typical for chemisorption. In the present study, the obtained ΔG^0^ ranged between −1 to −3 kJ/mol, meaning that the adsorption of BPA by GO is a physisorption process. The negative value of ΔH^0^ confirms that the adsorption reaction was exothermic. The negative value of ΔS^0^ implies a subsidence in randomness by the adsorbed species and indicates the stability of the adsorption process with no structural change at the solid–liquid interface [20]. The similar trend in the results was also observed for the BPA adsorption on graphene, reduced graphene oxide (rGO), and Fe_3_O_4_/GO [29,33,34].

### 2.3. Adsorption Kinetics

An adsorption kinetic study is generally considered for a better understanding of the reaction pathways and mechanisms of sorption reactions. This understanding could be applied to the proper design of adsorption systems. In the present study, the adsorption kinetics were fitted with the pseudo-first-order model and the pseudo-second-order model.

The pseudo-first-order model can be expressed as shown in Equation (3):

(3) $$\text{ln}\left(q_{e}-q_{t}\right)={\text{lnq}}_{e}-k_{1}t$$

where q_e_ and q_t_ are the amounts of BPA adsorbed on GO at equilibrium and at time interval t (mg/g), respectively, and k_1_ is the rate constant of pseudo-first-order adsorption (min^−1^). The values of q_e_ and k_1_ for the pseudo-first-order model were determined from the intercept and the slope of the linear plot of ln(q_e_ − q_t_) versus t, respectively.

The pseudo-second-order model is defined as shown in Equation (4):

(4) $$\frac{t}{q_{t}}=\frac{1}{k_{2}q_{e}^{2}}+\frac{t}{q_{e}}$$

where q_e_ and q_t_ are specified as in the pseudo-first-order model and k_2_ is the rate constant of the pseudo-second-order model for adsorption (g/mg/min). The slope and intercept of the linear plot of t/q_t_ against t yield the values of q_e_ and k_2_. Furthermore, the initial adsorption rate h (mg/g/min) can be determined from h = k_2_$q_{e}^{2}$.

**Figure 6:**
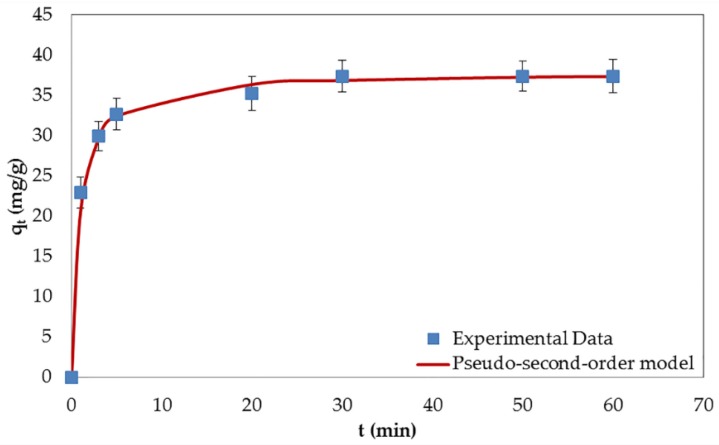
Adsorption kinetics of BPA on GO at the initial BPA concentration of 60 mg/L.

All parameters of the kinetic models are shown in Table 3. The pseudo-second-order model was proved to be dominantly fitted with the experimental data (see Figure 6). The reason can be ascribed to the former model’s higher correlation coefficient (R^2^), as well as to a better match of its adsorption capacity with the adsorption capacity calculated from the experimental data. The results show that the adsorption kinetics is divided into three stages. The first stage, named the film diffusion stage, demonstrates a fast diffusion of the BPA molecules from the solution onto the external surface (liquid film) of GO. The second stage, called the intraparticle diffusion stage, expresses the slow adsorption stage imputed to the penetration of the BPA molecules into the inner layer of liquid film. The third, and last, stage is the dynamic equilibrium stage, which is approached when the rate of adsorption equals the rate of desorption [35].

### 2.4. Adsorption Isotherms

An adsorption isotherm study describes the interactions between the absorbent and the adsorbate. It is crucial for optimization of the adsorption mechanism pathways, interpretation of the surface properties and adsorption capacities of adsorbents, and the effective design of the adsorption systems. To complete the adsorption isotherm study, the equilibrium data were fitted with the two common isotherm models, namely, the Langmuir isotherm and the Freundlich isotherm. The Langmuir isotherm model assumes monolayer adsorption on a homogeneous surface consisting of a limited number of identical sites. All sites are energetically equivalent and no interactions between adsorbed molecules occur. The Freundlich isotherm, in addition to the interactions between adsorbed molecules, describes the multilayer adsorption on heterogeneous surfaces [36]. The Langmuir isotherm model is expressed by Equation (5):

(5) $$\frac{C_{e}}{q_{e}}=\frac{C_{e}}{q_{m}}+\frac{1}{q_{m}K_{L}}$$

where C_e_ (mg/L) and q_e_ (mg/g) are the concentration of BPA and adsorption capacity at equilibrium, respectively; K_L_ (L/mg) is the Langmuir adsorption coefficient related to the energy of adsorption, and q_max_ (mg/g) is the maximum adsorption capacity. The values of q_m_ and K_L_ are consecutively calculated from the slope and intercept of the linear plot of C_e_/q_e_ against C_e_.

The Freundlich isotherm model is defined as shown in Equation (6):

(6) $${\text{lnq}}_{e}=\frac{\left({\text{lnC}}_{e}\right)}{n}+{\text{lnK}}_{F}$$

where K_F_ ((mg/g)/(mg/L)^1/n^) is the Freundlich adsorption coefficient and n is the dimensionless number related to the heterogeneity of the adsorption sites. K_F_ and n can be obtained from the intercept and slope of the linear plot of lnq_e_ versus lnC_e_, respectively.

**Figure 7:**
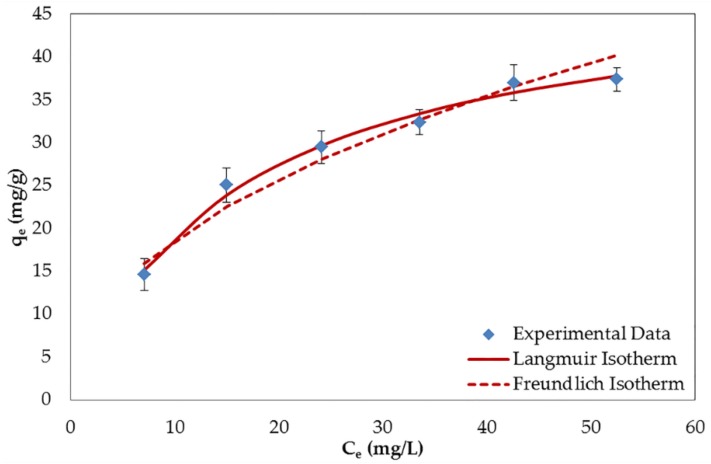
Adsorption isotherms of BPA on GO at a temperature of 298 K. C_e_: the concentration of BPA at equilibrium (mg/L).

**Figure 8:**
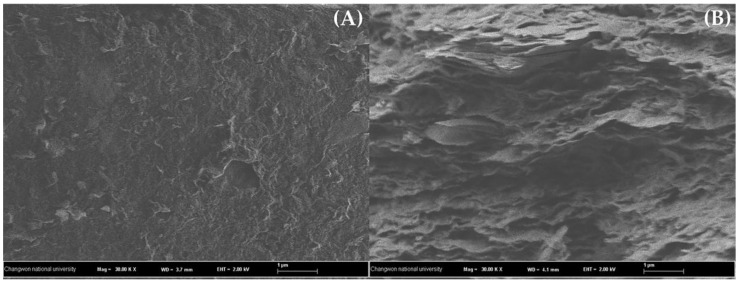
SEM images of (**A**) GO surface and (**B**) cross-section.

The isotherm parameters are listed in Table 4. Based on the comparison of the correlation coefficient (R^2^) values and the plots presented in Figure 7, the Langmuir isotherm model fitted the experimental data better than the Freundlich isotherm model. This means that the adsorption of BPA on GO was a monolayer adsorption on the homogeneous surface. Even though the data were described by the Langmuir model, the R^2^ values acquired from the Freundlich model are also significant (>0.95). The reasons for this finding may be assigned to the butterfly structure of the BPA molecule which creates a chance to form multilayer adsorption on the heterogeneous area [35]. The heterogeneous surface can refer to the aggregation, interstitial area, groove region, and some defection regions generated during the synthesis process [37,38]. In addition, the two hydroxyl groups in the BPA molecule may possibly cause the self-assembly of the adsorbate via hydrogen bonding which leads to the multilayer adsorption [39]. Since the adsorption mechanisms were better explained by the Langmuir isotherm, the monolayer adsorption was observed in major and the multilayer adsorption caused by the aforementioned reasons was observed in minor. The simultaneous occurrence of monolayer and multilayer adsorption can also be confirmed by the SEM images of GO (see Figure 8). The flat surface of GO in Figure 8A ascribes to the monolayer adsorption, while the multilayer adsorption may formed at the rough area of the cross-section (see Figure 8B). At 298 K, the maximum adsorption capacity (q_m_) calculated from the Langmuir isotherm amounted to 49.26 mg/g. This value is quite low as compared to other CNMs (graphene, rGO, and CNTs) that carry a lower percentage of OCFGs [33,34,40]. The reasons for this finding may be related to the complex interactions on the GO surface (see Section 3 for further discussion).

## 3. Discussion

**Figure 9:**
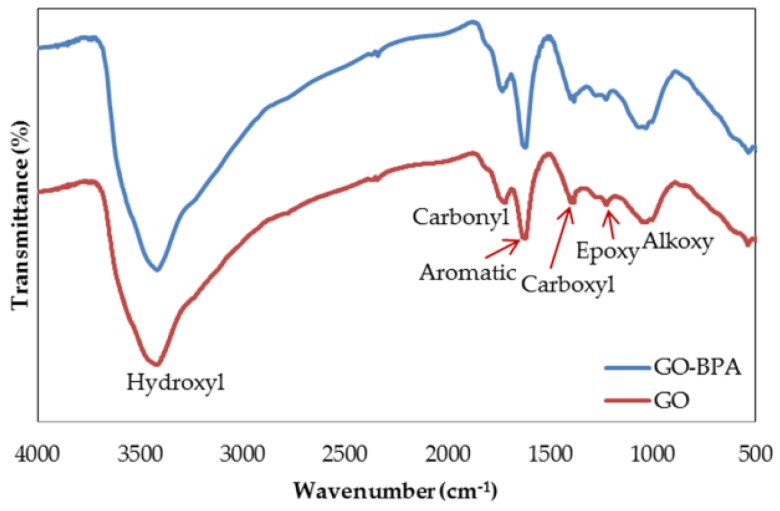
The oxygen-containing functional groups (OCFGs) on the GO surface analyzed by Fourier transform infrared spectroscopy (FTIR).

To classify the types of OCFGs on the GO surface, Fourier transform infrared spectroscopy (FTIR) analysis is necessary. The FTIR results of graphite and GO are illustrated in Figure 9. Each peak in FTIR results displays the appearance of different functional groups on the surface of the materials. Compared to graphite (as data referred from elsewhere), the peak intensities of GO are distinctly enhanced, demonstrating the success of oxidization. The cooperation among KMnO_4_, H_2_SO_4_, and H_3_PO_4_ destroys the π–orbital system of graphite and inserts diverse types of OCFGs into the carbon skeleton [41]. The obvious peak at 3419 cm^−1^ represents the existence of the hydroxyl group (O–H). The following peak at 1720 cm^−1^ is attributed to the carbonyl group (C=O). The formation of aromatic bonds (C=C) corresponds to the peak at 1619 cm^−1^. The evidence for the carboxyl group (O=C–OH) can be explained by the peak at 1384 cm^−1^. The next peak at 1227 cm^−1^ is considered as the epoxy group (C–O–C). The last peak at 1052 cm^−1^ is considered as the alkoxy group (C–O). These results are congruent with those reported in previous studies, which convincingly demonstrates the attainment of our synthesized GO [42,43].

**Figure 10:**
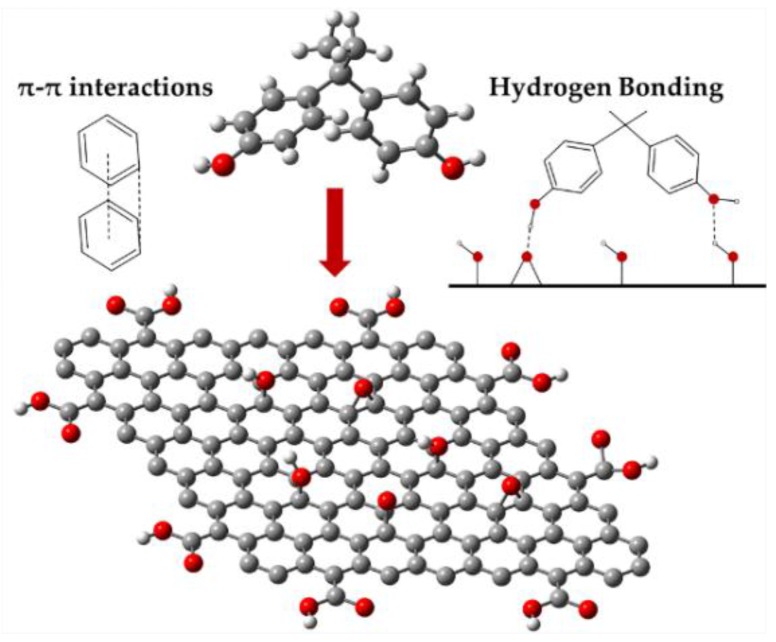
Schematic representation of π–π interactions and hydrogen bonding between BPA and GO.

After the adsorption of BPA, the peak intensities were modified in the whole range assigned to the interactions between BPA and GO. The interactions between BPA and OCFGs were affirmed by the change of peaks from 3419 cm^−1^, 1720 cm^−1^, 1227 cm^−1^, 1052 cm^−1^ to 3418 cm^−1^, 1733 cm^−1^, 1228 cm^−1^, and 1065 cm^−1^, respectively. These moves may represent the hydrogen bonding formed between the hydroxyl groups of BPA and OCFGs of GO. As evidenced by the shift of the band from 1619 cm^−1^ to 1623 cm^−1^, π–π interactions also played a role in the adsorption. These interactions existed between the benzene rings of both the adsorbate and the adsorbent. The FTIR result reveals that the adsorption of BPA onto GO is held by both hydrogen bonding and π–π interactions. The schematic of both adsorption interactions is shown in Figure 10. The appearance of OCFGs appears to be helpful for adsorption. This result is also supported by the analogous structures of the adsorbate and the adsorbent. The structure of BPA consists of hydrophilic hydroxyl groups and hydrophobic benzene rings. This amphiphilic property is similar to GO which contains hydrophilic functional groups and a hydrophobic basal plane. The compatibility of properties in the adsorbate and the adsorbent may lead to a strong adsorption [35]. Additionally, since the length of BPA molecule is 0.94 nm and the average pore size of GO varies in the range from 2.3 to 5 nm, this pore size is sufficient for the BPA molecules to enter and be adsorbed on the adsorption sites [28,44,45]. The high performance of GO is confirmed not only by these explanations, but also by some previous computational studies [9,10]. The adsorption energy of BPA onto GO obtained by the DFT study is higher than that onto graphene. The adsorption of GO regarding the collaboration between hydrogen bonding and π–π interactions is shown to be stronger than the adsorption by only π–π interactions of graphene [9]. This confident evidence suggests that GO is the most suitable adsorbent for the removal of BPA as compared to other CNMs that contain a lower proportion of OCFGs. By contrast, the efficiency of GO for the removal of some aromatic pollutants that are free from positive charge was found to be the lowest among CNMs in previous experimental studies [11]. The reason behind this ineffectiveness may point to a large number of functional groups on the surface. This reason was investigated by Wang et al. [11] who studied the correlation between the reduction degree of GO and the adsorption efficiency of phenolic compounds. After the reduction of GO, the increment of π–π interactions alongside with adsorption performance was found. In other words, a lower number of OCFGs results in a higher adsorption efficiency. This means that π–π interactions are dominant interactions for the adsorption of phenolic compounds. Thus, the occurrence of OCFGs appears to hinder the potential of π–π interactions. The interpretations in both computational studies and experimental studies are definitely mutually contradictory.

**Figure 11:**
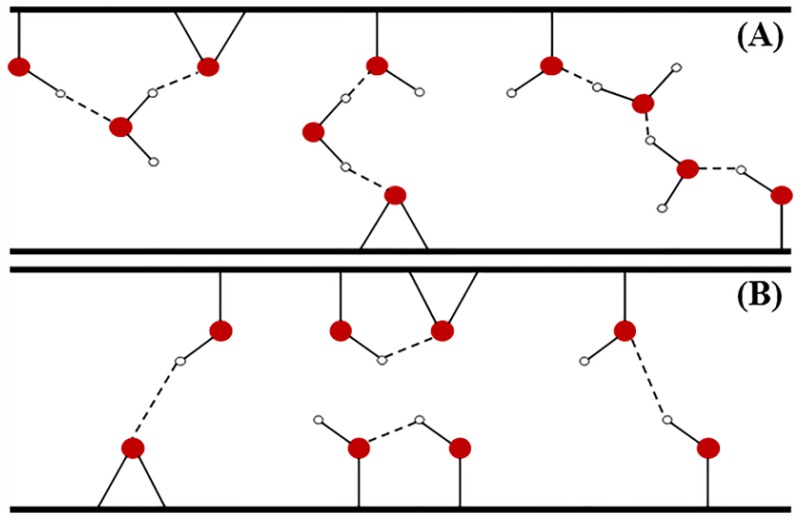
Hydrogen bonding: (**A**) OCFGs–H_2_O; and (**B**) OCFGs–OCFGs.

In our study, the maximum adsorption capacity of BPA onto GO amounted to 49.26 mg/g. This capacity is quite low as compared to other CNMs, according to previous studies (81.3–96.2 mg/g for rGO, 71–111 mg/g for CNTs, and 182 mg/g for graphene) [33,34,40]. Among CNMs, GO contains the highest number of OCFGs, followed by rGO, CNTs, and graphene. Thus, the adsorption capacity seems to increase with decreasing the number of OCFGs. Our results support the inference of other experimental studies that showed that GO is an ineffective material for the removal of BPA. Thus, the logical clarification is necessary in order to enhance the reliability of this summary. The possible issue may relate to the interference of water molecules. The competitive adsorption of a solvent is a common phenomenon in the solid–liquid adsorption system [35]. In aqueous solution, the adsorption may be hindered by the formation of hydrogen bonding between water molecules and OCFGs (see Figure 11A). Additionally, OCFGs can possibly bond with other surrounding functional groups located in the same GO sheet or in the neighboring sheets (see Figure 11B). These unwanted H-bonds have a large-scale effect on the adsorption efficiency of GO by sealing the high-adsorption-energy binding sites. Instead of enhancing the efficiency, the occurrence of OCFGs has a negative effect on the adsorption by reducing the efficiency and obstructing the formation of π–π interactions, which is supposed to be the main interaction for the adsorption on CNMs. Accordingly, GO that contains a high proportion of OCFGs seems to be critically affected by these phenomena. Compared to other CNMs, these effects are almost meaningless [46]. The conclusion was also declared by several molecular dynamics studies that demonstrated a plentiful hydrogen bonding between OCFGs and water molecules, as well as the intra/inter-layer H bonds among OCFGs on the GO surface [47,48]. In computational studies revealing the suitability of GO for the BPA adsorption, the DFT studies were performed without considering the aforementioned issues. Even though the adsorption energy was found to be higher in GO, this investigation cannot be used to illustrate the adsorption behavior in reality. Up to now, our study has clearly emphasized that the efficiency of GO for adsorption of BPA in water is low. Thus, some specific modifications on the GO surface should be applied in order to improve its adsorption performance. A better efficiency after modification of adsorbents was confirmed in several previous studies [37,49,50].

## 4. Materials and Methods

### 4.1. Materials

Graphite powder (500 mg) was purchased from Kanto Chemical*.* Co., Inc, (Chuo-ku, Tokyo, Japan)*.* Other chemicals (99% BPA, KMnO_4_ flake, 98% H_2_SO_4_, 85% H_3_PO_4_, 34.5% H_2_O_2_, 35% HCl, Acetone, and deionized (DI) water) were ordered from Daejung Chemical and Metals Co., Ltd. (Siheung-city, Gyonggi-do, Korea). The BPA stock solution (60 mg/L) was prepared in DI water by shaking overnight without mixing with other chemicals to avoid the co-solvent effect. The stock solution was further diluted to the required concentrations before use.

### 4.2. Synthesis of Graphene Oxide

#### 4.2.1. Oxidation of Graphite

GO was synthesized following the Improved Hummers’ method [51]. A 9:1 mixture of concentrated H_2_SO_4_/H_3_PO_4_ (360:40 mL) was added to a 1:6 mixture of graphite powder/KMnO_4_ (3:18 g). In accordance with the intense exothermicity of this reaction, both mixtures need to be cooled in a fridge before mixing to avoid an explosion due to the occurrence of the strong oxidant Mn_2_O_7_. This oxidant is known to detonate at the temperature over 55 °C [52]. The exothermic reaction of mixing will heat up the temperature of solution to 35–40 °C. The reaction was then heated to 50 °C and stirred for 12 h by using a magnetic stirrer to ensure the complete oxidation reaction of graphite. The mixture was then cooled down to room temperature and poured onto ice (made from 400 mL of DI water) followed by adding 3 ml of 30% H_2_O_2_ to reduce the residual permanganate and manganese dioxide [53]. The bright yellow solution was received as the final product.

#### 4.2.2. Washing the Oxidized Graphite

The final solution was repeatedly washed to remove the byproducts (e.g., potassium-containing compounds) that can cause an acute explosion and combustion [54]. The washing process was started with the filtering of the mixture through a 45 µm testing sieve (Chung Gye Sang Gong Sa, Seoul, Korea), followed by the centrifugation of the filtrate at 4000 rpm for 3 h; then, the supernatant was decanted away. The remaining settling was then washed in succession with 200 mL of DI water, 200 mL of 30% HCl (2 times), and 200 mL of Acetone (five times) [54]. For each wash, the mixture was again filtered through the testing sieve and centrifuged as mentioned above. After the washing process, the color of sediment turned to dark brown. Finally, the product was filtered over the polytetrafluoroethylene (PTFE) membrane with a pore size of 0.45 µm. The GO paste attached to the filter was vacuum-dried overnight at room temperature, which let us obtain 5.8 g of the GO powder. The particle size of the GO powder was controlled to be below 0.2 mm.

### 4.3. Characteristic Analysis of the Synthesized Graphene Oxide

The synthesized GO was characterized by powder X-ray diffraction (XRD) on a Bruker D8-advance X-ray diffractometer (Bruker AXS GmbH, Karlsruhe, Germany) at 40 kV and 40 mA for monochromatized Cu *K*α (λ = 0.1541 nm) radiation. The XRD data were collected in a 2θ range from 5° to 70°. The Fourier transform infrared (FTIR) spectra were recorded on an FTIR-6300 (JASCO, Hachioji, Tokyo, Japan spectrometer in the transmittance mode. The range of the detectable signal was 4000–400 cm^−1^ with the resolution of 4 cm^−1^. The morphology energy-dispersive X-ray spectroscopy (EDS) of both samples was inspected using a scanning electron microscope (SEM, JSM-6510, JEOL Ltd., Akishima, Tokyo, Japan) equipped with an energy-dispersive X-ray analyzer (EDX, Oxford INCA, Oxford Instruments, Abingdon, Oxfordshire, UK).

### 4.4. Batch Adsorption Experiments

To clarify the adsorption characteristics, batch experiments for adsorption kinetics, isotherms including the effects of various parameters are necessary. Each experiment was performed using a series of volumetric flasks containing 50 mL of the BPA solution. The experimental conditions in each experiment are specified in Table 5. The samples were shaken in a shaking incubator (JEIO TECH, SI-900R, Geumcheon-gu, Seoul, Korea) with constant agitation (200 rpm).

After the adsorption experiments, the samples were centrifuged at 5000 rpm for 20 min to separate GO from the samples. The supernatants were taken out and centrifuged again at 13,000 rpm for 20 min to remove the residual GO in the samples. The BPA concentration in the samples was measured by high-performance liquid chromatography (HPLC, Shimadzu, Kyoto, Japan) system with a 150 mm × 4.6 mm, 3.5 µm Agilent Zorbax SB-C18 column (Agilent, Santa Clara, CA, USA). The UV-VIS detector (SPD-20A, Shimadzu, Kyoto, Japan) was operated at 280 nm. The mobile phase was 1.0 mL/min of 70% methanol and 30% deionized water. To warrant the accuracy of the results, the experiments were completed at least three times. The adsorption capacity (mg/g) was calculated using Equation (7):

(7) $$q_{e}=\frac{\left(C_{0}-C_{e}\right)V}{m}$$

where C_0_ and C_e_ are the concentrations of BPA in the solution (mg/L) at the initial stage and at equilibrium, respectively; V is the volume of solution (L); and m is the adsorbent mass (g).

## 5. Conclusions

In the present study, the adsorption characteristics of BPA on GO in aqueous solution were investigated. The adsorption reached equilibrium within 30 min, indicating the rapid adsorption of BPA on GO. The increment of adsorption capacity after increasing the initial BPA concentration could be illustrated by the support of the driving force which overcame the mass transfer resistance between the aqueous and the solid phases. The reverse result was obtained after increasing the adsorbent dosage. This pattern can be explained by the aggregation and overlapping of the adsorbent particles that obstructed the adsorption. By varying the pH of the solution, the adsorption efficiency was constantly high in the acidic pH range, but dramatically decreased when the pH increased above 8. This trend could be explained by the deprotonation of the BPA molecules that cause the electrostatic repulsion with the GO surface. The adsorption process proved to be exothermic and spontaneous, according to the thermodynamic study. The adsorption kinetics of BPA on GO could be described by the pseudo-second-order model covering the three stages of adsorption, including film diffusion, intraparticle diffusion, and the equilibrium stage. The equilibrium sorption of BPA could be properly fitted by both the Langmuir and the Freundlich isotherms, as suggested by the existence of both monolayer and multilayer adsorption. It may be explained by the nature of BPA molecules and the defect of the GO surface. By consulting the results of other experimental and computational studies, the BPA adsorption onto GO in the aqueous solution was demonstrated to be driven by π–π interactions, rather than by hydrogen bonding. The appearance of OCFGs on GO was found to hinder the adsorption for two reasons. First, the possibility of formation of hydrogen bond between the water molecules and OCFGs will create competition for adsorption of the adsorbate. Second, hydrogen bonding also can be formed among OCFGs on the GO surface by bonding within the GO sheet (intra-layer H-bonds) and by bonding with the nearby sheets (inter-layer H-bonds). These effects should be considered in further computational studies in order to achieve a clear and reliable understanding of the adsorption mechanisms. As it becomes obvious from the discussion above, even though the use of GO is ineffective for the BPA adsorption, suitable modifications of the GO surface may lead to further development of a novel adsorbent that is appropriate for the removal of BPA.

## Figures and Tables

**Table 1:** Comparison of the elemental analysis of graphene oxide (GO) measured by scanning electron microscopy with energy dispersive X-ray spectroscopy (SEM/EDS) with the results of previous studies.

Adsorbent	Weight %			Atomic %			Atomic ratio (C/O)	References
	C	O	Other	C	O	Other		
GO	49.44	40.99	9.57	58.76	36.58	4.66	1.61	(this study)
	58.36	36.85	4.79	66.45	31.50	2.05	2.11	[12]
	-	-	-	59.3	31.4	9.3	1.89	[13]
	-	-	-	58	38	4	1.53	[14]
	-	-	-	53.32	43	3.68	1.24	[15]

**Table 2:** Thermodynamic parameters for the adsorption of BPA by GO.

Temperature (K)	K_d_	ΔG^0^ (kJ/mol)	ΔH^0^ (kJ/mol)	ΔS^0^ (J/mol/K)
283	3.479	−3.122	−18.646	−54.854
298	2.995	−2.300	−18.646	−54.854
313	1.614	−1.476	−18.646	−54.854

**Table 3:** Kinetic parameters for the adsorption of BPA by GO.

q_e,exp_ (mg/g)	Pseudo-First-Order			Pseudo-Second-Order			
	k_1_ (1/min)	q_e,cal_ (mg/g)	R^2^	k_2_ (g/mg/min)	q_e,cal_ (mg/g)	h (mg/g/min)	R^2^
25.074	0.0699	3.526	0.865	0.1042	25.126	65.789	0.9999
37.379	0.1417	15.206	0.981	0.0315	37.879	45.249	0.9998

**Table 4:** Isotherm parameters for the adsorption of BPA by GO at different temperatures.

Temperature (K)	Langmuir			Freundlich		
	q_m_ (mg/g)	K_L_ (L/mg)	R^2^	K_F_	n	R^2^
283	62.893	0.0534	0.9907	7.116	2.054	0.9831
298	49.261	0.0627	0.9939	6.421	2.158	0.9569
313	32.895	0.0495	0.9910	3.580	2.047	0.9809

**Table 5:** Experimental conditions.

Parameters	Contact Time (min)	Initial BPA Concentration (mg/L)	GO Dosage (mg)	pH	Temperature (K)
Contact time	1–60	60	10	3.5 ± 0.5	298
Initial BPA concentration	1–60	20, 60	10	3.5 ± 0.5	298
GO dosage	120	60	10–200	3.5 ± 0.5	298
pH	120	60	10	3–12	298
Temperature	120	60	10	3.5 ± 0.5	283–313
Kinetics	1–60	60	10	3.5 ± 0.5	298
Isotherms	120	10–60	10	3.5 ± 0.5	298

## Abbreviations

The following abbreviations are used in this manuscript:

BPA	Bisphenol A
CNMs	Carbon nanomaterials
CNTs	Carbon nanotubes
DFT	Density functional theory
DI	Deionized
EDCs	Endocrine disrupting compounds
EDS	Energy dispersive X-ray spectroscopy
FTIR	Fourier transform infrared spectroscopy
GO	Graphene oxide
OCFGs	Oxygen-containing functional groups
PTFE	Polytetrafluoroethylene
rGO	reduced graphene oxide
SEM	Scanning electron microscopy
XRD	X-ray diffraction
